# p53 Binding Sites in Long Terminal Repeat 5Hs (LTR5Hs) of Human Endogenous Retrovirus K Family (HML-2 Subgroup) Play Important Roles in the Regulation of LTR5Hs Transcriptional Activity

**DOI:** 10.1128/spectrum.00485-22

**Published:** 2022-07-18

**Authors:** Mengying Liu, Lei Jia, Hanping Li, Yongjian Liu, Jingwan Han, Xiaolin Wang, Tianyi Li, Jingyun Li, Bohan Zhang, Xiuli Zhai, Changyuan Yu, Lin Li

**Affiliations:** a College of Life Science and Technology, Beijing University of Chemical Technology, Beijing, China; b Department of Virology, Beijing Institute of Microbiology and Epidemiology, Beijing, China; c State Key Laboratory of Pathogen and Biosecurity, Beijing, China; University of Florida

**Keywords:** HERV, HML-2, LTR5Hs, p53, transcriptional activity

## Abstract

The long terminal repeats (LTRs) of human endogenous retroviruses (HERVs) are distributed throughout the human genome and provide favorable conditions to regulate the expression of their adjacent genes. HML-2 is the most biologically active subgroup of the HERV-K family, and expression of its members has been associated with many cancer types. The LTRs of HML-2 have been classified into three subgroups (LTR5A, LTR5B, and LTR5Hs) based on phylogenetic analyses. The current study aimed to explore the LTR transcriptional activity differences among the three subtypes and further explore the underlying factors. A total of 43 LTR5A elements, 62 LTR5B elements, and 194 LTR5Hs elements were selected. A phylogenetic tree showed that the LTR5Hs group was clearly separated from the LTR5A and LTR5B groups. A luciferase reporter assay indicated that LTR5Hs had the strongest promoter activity, followed by LTR5A and LTR5B. To investigate the underlying factors, LTR5Hs was divided into 4 sections, and the homologous fragments in LTR5B were replaced successively. Replacement of the third section (−263 to 0) significantly increased LTR5B activity. Subsequent mutation experiments revealed that the increased transcriptional activity was induced by the TATA box and the two p53 binding sites within the section. Further interference with *TP53* significantly decreased LTR5Hs transcriptional activity. Chromatin immunoprecipitation (ChIP) and CUT&Tag experiments finally confirmed the direct binding of the p53 protein with the two LTR5Hs p53 binding sites. Overall, the two p53 binding sites in the third section (−263 to 0) of LTR5Hs were revealed to play critical roles in the difference in transcriptional activity among the three subtypes.

**IMPORTANCE** Human endogenous retroviruses (HERVs) were integrated into the human genome in ancient times and have been coevolving with the host. Since the Human Genome Project, HERVs have attracted increasing attention. Many studies have focused on their characterization, evolution, and biological function. In particular, the expression of HERV-K has been associated with many diseases, such as germ cell tumors, neurotoxicity, ovarian cancer, prostate cancer, and melanoma. Indeed, two HML-2-produced proteins, Np9 and Rec, are associated with certain cancers. However, their roles in these disease associations remain unclear. The current work focused on subgroup HML-2 of HERV-K, which is recognized as the most biologically active subgroup, and aimed to explore the mechanistic basis of transcriptional activity. The results revealed that p53 deeply determined the activity of HML-2 LTR5Hs. p53 is a rather important tumor suppressor protein. It can regulate the expression of genes related to cell cycle arrest, organic processes, and apoptosis in response to cellular stress and is critical for the control of homeostasis. Previous ChIP and expression studies of individual genes suggested that p53 sites in HERV LTRs may be part of the p53 transcription program and directly regulate p53 target genes in a species-specific manner. However, the exact function of p53 in the regulation of HERV LTR expression is largely elusive. Our results clearly demonstrated the interaction between LTR5Hs of HML-2 and p53. They are of great significance for the future comprehensive study of the physiological and pathological functions of LTRs of HERVs.

## INTRODUCTION

Human endogenous retroviruses (HERVs), which stem from exogenous retrovirus infection during the evolution of primates and subsequent integration into the genome, account for approximately 8% of the human genome (1–3). The vast majority of HERVs lack infectious capacity due to accumulation of deletions, mutations, and insertions of internal coding regions and long terminal repeats (LTRs) (2). HERVs have been divided into three classes: class I, which consists of gamma retrovirus-like elements; class II, which consists of beta retrovirus-like elements; and class III, which consists of vaguely spumaretrovirus-like elements (4). HML-2 of HERV-K, the clade of beta retrovirus-like endogenous retroviruses, is recognized as the most biologically active subgroup, and many of its members retain transcriptional activity (2, 5–8). Expression of these members has been associated with many diseases, such as germ cell tumors, ovarian cancer, prostate cancer, melanoma, rheumatoid arthritis, and amyotrophic lateral sclerosis (ALS) (6, 9–11).

A complete HML-2 provirus is approximately 9.5 kb in length and consists of four overlapping genes that encode structural and nonstructural proteins, with an LTR structure (5′ LTR-gag-pro-pol-env-3′ LTR) at each terminus (5, 12). Each flanking viral LTR consists of U3, R, and U5 regions in the 5′-to-3′ direction; these regions possess promoter elements, enhancer elements, TATA-independent promoters, polyadenylation signals, and multiple transcription factor-binding sites (TFBSs) (13–15).

Based on phylogenetic analyses, the LTR sequences of HML-2 are classified into three subgroups: LTR5A, LTR5B, and LTR5Hs. LTR5B is the oldest ancestral type, while LTR5Hs is the most recently acquired. LTR5A and LTR5Hs were independently generated from LTR5B (2, 6, 13, 16, 17).

HERV-K (HML-2) LTRs exist as part of complete provirus structures or as single-LTR structures called solo LTRs (16). Within the entire human genome, there are more than 1,000 HERV-K (HML-2) loci. Most of them are solo LTRs, which are produced by homologous recombination between the LTRs of a single HERV-K (HML-2) (5, 18). Solo LTRs are approximately 10-fold more abundant than their full-length or nearly full-length proviral integrations (2). LTRs can be inserted into many areas of the host chromosome, including introns, exons, and intergenic regions. At least 50% of human-specific LTRs have promoter activity and are located in the sense and antisense orientations of DNA (19–21). These distributions provide favorable conditions for LTRs to regulate the expression of their adjacent genes (20). Genome-wide analysis results have revealed the important role of HERV-K in the construction of gene regulatory networks through the functional TFBSs possessed in LTR sequences (22, 23).

Although the functions of many transcription factors have been revealed, it is unclear whether the transcriptional activities of the three HML-2 LTR subtypes are different, and the LTR TFBSs associated with HERV function are largely elusive. In this study, we investigated the transcriptional activity differences among LTR5A, LTR5B, and LTR5Hs of HML-2 and explored the key TFBSs.

## RESULTS

### Localization and characterization of HML-2 LTR5A, LTR5B, and LTR5Hs sequences.

Based on BLAT (BLAST-Like Alignment Tool) analysis using Dfam consensus representatives, a total of 704 LTR5A sequences, 828 LTR5B sequences, and 256 LTR5Hs sequences were identified (see Table S1 in the supplemental material). After alignment using ClustalW in BioEdit, we screened 43 LTR5A sequences, 62 LTR5B sequences, and 194 LTR5Hs sequences, which were longer than 60% of the Dfam full-length references for each type, for further analyses. The majority of sequences in the data set covered the third fragment (−263 to 0), which was the target region of the current work. The identified sequences and reference sequences used to construct the phylogenetic trees are shown in Table S2.

### Phylogenetic analyses.

**FIG 1:**
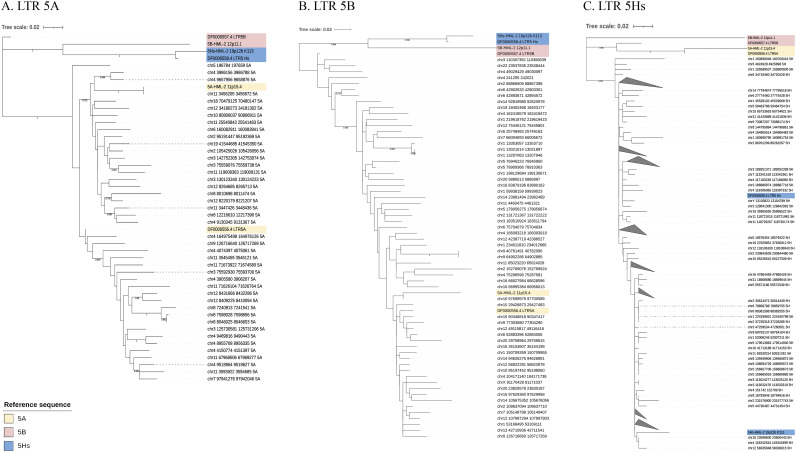
Phylogenetic analysis of HML-2 LTR5A, LTR5B, and LTR5Hs. The screened HML-2 elements of LTR5A (A), LTR5B (B), and LTR5Hs (C) were analyzed with the ML method. The reference sequences are marked in different colors. The resulting phylogeny was tested by using the bootstrap method with 1,000 replicates. Bootstrap values of >0.70 (0 to 1) are shown for the best rooted tree.

To characterize the phylogenetic relationships among HML-2 LTR5A, LTR5B, and LTR5Hs, the 43 LTR5A sequences, 62 LTR5B sequences, 194 LTR5Hs sequences, and reference sequences were used to construct maximum-likelihood (ML) trees. Overall, our classification was the same as those of the previous reports defining the three major subgroups (2). As shown in Fig. 1A and C, the LTR5Hs group was clearly separated from the LTR5A and LTR5B groups. In addition, the LTR5A subgroup contained a clade comprising two clusters, supported by bootstrap values of 0.752 and 0.896. As shown in Fig. 1B, LTR5A sequences were nested within the LTR5B sequences in the tree. Previous studies similarly revealed that HML-2 LTRs clustered into three subgroups based on phylogeny: LTR5A, LTR5B, and LTR5Hs (13, 17). Our tree topology is consistent with these previous reports (13, 17, 24, 25) (Fig. 1). LTR5A should not be a distinct cluster given that LTR5A sequences were nested within the LTR5B sequences (2). Rather, it should be assigned to the LTR5B cluster. Thus, there is a need to reclassify the LTR subgroups. Overall, these results support the theory that the LTR5B subgroup is the oldest and most ancestral, while LTR5A and LTR5Hs independently originated from the LTR5B group (2).

### Functional prediction of *cis*-regulatory regions.

**FIG 2:**
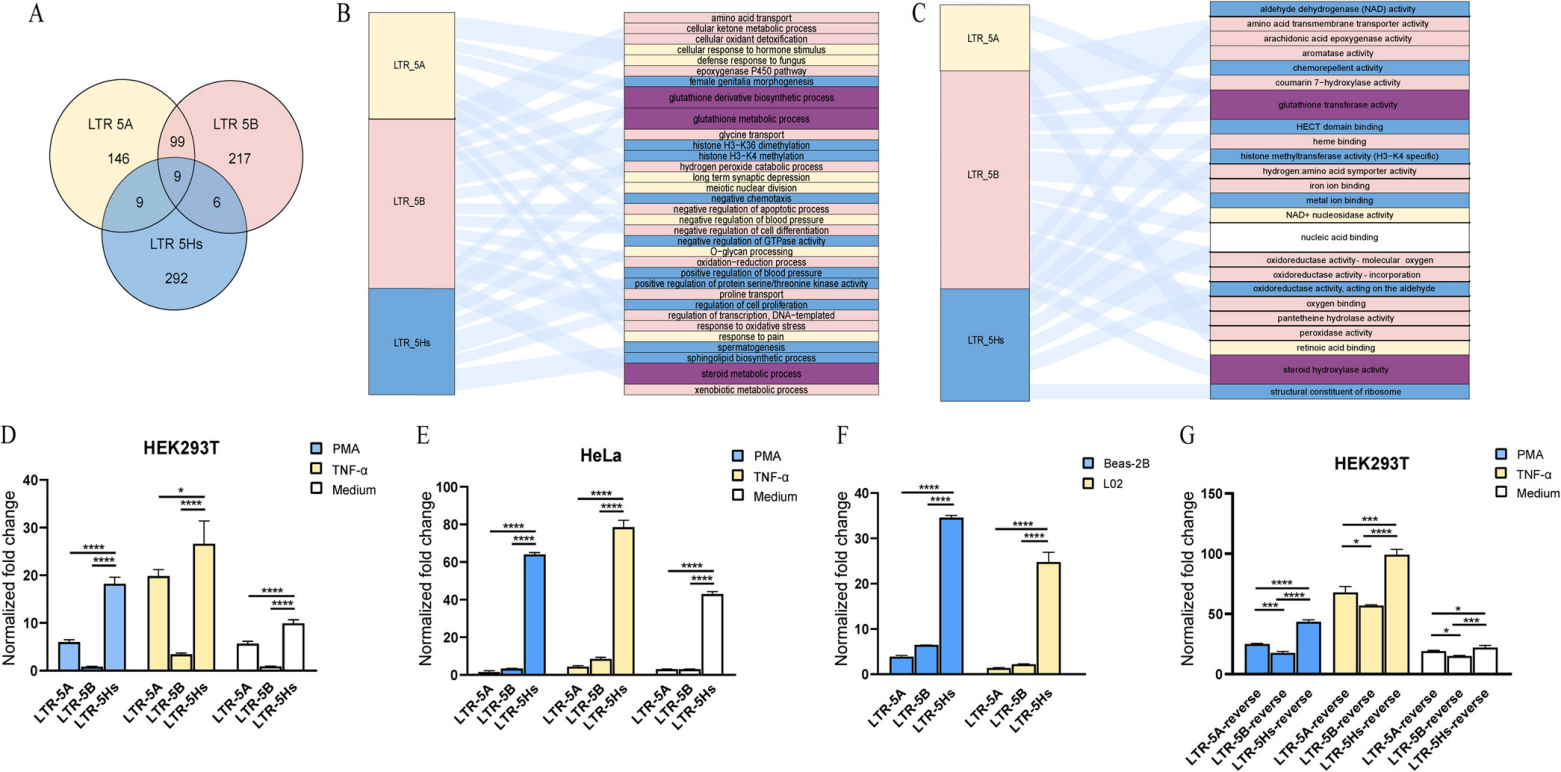
Analysis of the regulated genes and transcriptional activity of LTR5A, LTR5B, and LTR5Hs. (A) Venn diagram showing the numbers of regulated genes. (B and C) Sankey diagrams for the BPs (B) and MFs (C) of LTR5A-, LTR5B-, and LTR5Hs-regulated genes. Notably, the results are entirely speculative based on GREAT and DAVID and have not yet been proven. The three different subtypes and their related BPs/MFs are marked with yellow, pink, and blue. Purple represents the common BPs/MFs of LTR5A and LTR5B, whereas white indicates the common BPs/MFs of LTR5B and LTR5Hs. (D to F) Promoter activity of LTR5A, LTR5B, and LTR5Hs. Promoter activity values of LTR5A, LTR5B, and LTR5Hs in the HEK293T (D) and HeLa (E) cell lines. LTR5A, LTR5B, and LTR5Hs luciferase reporter plasmids and pRenilla-luc-TK were cotransfected into HEK293T and HeLa cells for 24 h, and cells were stimulated with TNF-α or PMA-ionomycin for an additional 24 h. (F) Promoter activity of LTR5A, LTR5B, and LTR5Hs in Beas-2B and L02 cells. LTR5A, LTR5B, and LTR5Hs luciferase reporter plasmids and pRenilla-luc-TK were cotransfected into Beas-2B and L02 cells for 48 h. (G) Promoter activity of the reverse sequences of LTR5A, LTR5B, and LTR5Hs in HEK293T cells. The reverse sequences of LTR5A, LTR5B, and LTR5Hs luciferase reporter plasmids and pRenilla-luc-TK were cotransfected into HEK293T cells for 24 h, and cells were stimulated with TNF-α or PMA-ionomycin for an additional 24 h. The normalized fold change was obtained with the formula shown in Materials and Methods. Error bars indicate standard errors for the results of three independent experiments. *, *P* < 0.05; ***, *P* < 0.001; ****, *P* < 0.0001 (one-way analysis of variance [ANOVA]).

Typically, noncoding regions lack biological function annotations. To study the biological significance of LTR5A, LTR5B, and LTR5Hs in the human genome, the putative *cis*-regulatory roles of these LTR elements were predicted with the Genomic Regions Enrichment of Annotations Tool (GREAT), which can predict genes potentially regulated by these elements based on spatial proximity. In total, 263, 331, and 316 potentially regulated genes were obtained for LTR5A, LTR5B, and LTR5Hs, respectively. The gene names and distances from LTR elements are shown in Table S3. The results showed that there was little overlap among the three subtypes (Fig. 2A). Only 9 genes (*KMT2E*, *PABPC5*, *GSTA4*, *HIST1H2BL*, *GSTA3*, *FAM218A*, *OR11H4*, *TRIM61*, and *ZNF184*) were predicted to be regulated by all three subtypes (LTR5A, LTR5B, and LTR5Hs). Ninety-nine genes were predicted to be regulated by both LTR5A and LTR5B, 9 genes were predicted to be regulated by both LTR5A and LTR5Hs, and 6 genes were predicted to be regulated by both LTR5B and LTR5Hs. The results are based on the potential regulatory genes predicted by GREAT according to spatial proximity. Further research is required to confirm any of the implied associations between the solo LTRs and the nearby genes.

We further analyzed the Gene Ontology (GO) enrichment results of the genes predicted to be regulated by the 3 types (Table S4). The results showed that the genes potentially regulated by LTR5A, LTR5B, and LTR5Hs were involved in almost completely distinct biological processes (BPs) and molecular functions (MFs) (Fig. 2B and C). Many of the genes that can be regulated by both LTR5A and LTR5B are associated with the glutathione derivative biosynthetic process, glutathione metabolic process, and steroid metabolic process. While many of the genes that can be regulated by both LTR5B and LTR5Hs are linked to nucleic acid binding molecular functions. Since the three subtypes had very different predicted regulated genes, we assume that LTR5A, LTR5B, and LTR5Hs have different activities in the genome and regulate their specific target gene populations through specific transcription factors. Notably, these results are entirely speculative; future research is needed.

### LTR5A, LTR5B, and LTR5Hs of HML-2 have different promoter activities.

To investigate the potential differences in the promoter activity among the 3 HML-2 LTR subtypes, we screened three representative strains (2) and synthesized the LTRs of HML-2_11p15.4 (1,027 bp), HML-2_12p11.1 (983 bp), and HML-2_19p12b_K113 (967 bp) as reference sequences for LTR5A, LTR5B, and LTR5Hs, respectively. These LTR sequences and their reverse sequences were cloned into the luciferase-based reporter vector pGL4.17 and transfected into the HEK293T, HeLa, Beas-2B, and L02 cell lines. Then, the luciferase activity levels were measured under different stimuli (tumor necrosis factor alpha [TNF-α], phorbol 12-myristate-13-acetate [PMA]/ionomycin, or cell culture medium only). The results showed that LTR5Hs possessed the strongest promoter activity in all cell lines under different stimulations. In comparison, LTR5A and LTR5B displayed a significantly lower promoter activity (Fig. 2D to F). The reverse sequences of LTR5A, LTR5B, and LTR5Hs showed the same trend, but the promoter activity of the reverse sequence of LTR5B was much higher than that of the forward sequence (Fig. 2G). In addition, a luciferase assay was performed in the A549, HepG2, MT-2, and Jurkat T cell lines. Similarly, the results showed that LTR5Hs possessed the strongest promoter activity in all cell lines (Fig. S1).

### The −263-to-0 fragment of LTR5Hs containing enhancer/core promoter sequences determines the difference in LTR activity.

**FIG 3:**
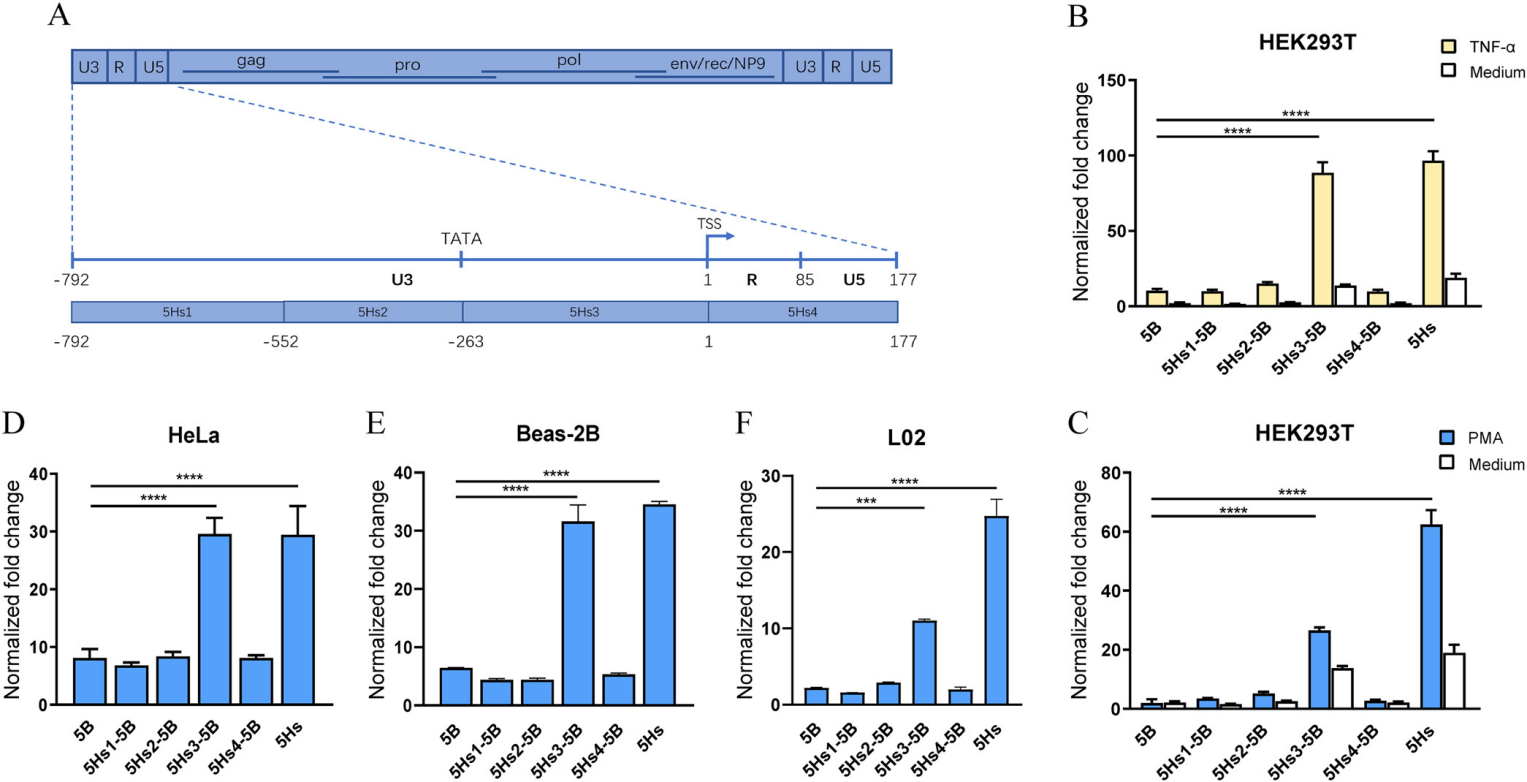
The −263 to 0 fragment of LTR5Hs determines LTR5Hs activity. (A) HERV provirus and LTR structure. The homologous fragments of LTR5B were replaced as depicted in the schematic. (B to F) Promoter activity of 4 sections in different cell lines. Promoter activity of 5Hs1-5B (−792 to −553), 5Hs2-5B (−552 to −264), 5Hs3-5B (−263 to 0), and 5Hs4-5B (1 to 177) in HEK293T cells stimulated with TNF-α (B) and PMA-ionomycin (C). 5Hs1-5B, 5Hs2-5B, 5Hs3-5B, and 5Hs4-5B luciferase reporter plasmids with pRenilla-luc-TK were cotransfected into HEK293T cells for 24 h, and cells were stimulated with TNF-α or PMA-ionomycin for an additional 24 h. Luciferase activity of the 4 modified LTRs in HeLa cells (D), Beas-2B cells (E), and L02 cells (F). 5Hs1-5B, 5Hs2-5B, 5Hs3-5B, 5Hs4-5B luciferase reporter plasmids, with pRenilla-luc-TK were cotransfected into cells for 48 h. The normalized fold change was obtained with the formula shown in Materials and Methods. Error bars indicate standard errors for the results of three independent experiments. ***, *P* < 0.001; ****, *P* < 0.0001.

To investigate which region determines the significantly high activity of LTR5Hs, LTR5Hs was divided into 4 sections. The homologous fragments in LTR5B were successively replaced to generate 5Hs1-5B (−792 to −553), 5Hs2-5B (−552 to −264), 5Hs3-5B (−263 to 0), and 5Hs4-5B (1 to 177) constructs (Fig. 3A). The 4 constructed plasmids with replaced sections and pRenilla-luc-TK were cotransfected into HEK293T cells for 24 h, and the cells were then stimulated with PMA-ionomycin, TNF-α, or cell culture medium for an additional 24 h. Luciferase activity was quantified by using a dual-luciferase reporter assay. The results revealed that replacement with 5Hs3-5B but not the 5Hs1-5B, 5Hs2-5B, or 5Hs4-5B region of LTR5Hs significantly increased LTR5B activity upon PMA-ionomycin, TNF-α, or cell culture medium treatment in HEK293T cells (Fig. 3B and C). To confirm these results, the 4 plasmids with replaced sections and pRenilla-luc-TK were cotransfected into three other cell lines (HeLa, Beas-2B, and L02), and luciferase activity was measured 48 h after transfection. The same result was obtained: 5Hs3-5B activity was significantly increased (Fig. 3D to F). Taken together, these data demonstrate that the third section (nucleotides −263 to 0) of LTR5Hs is responsible for the enhanced promoter activity of LTR5Hs.

### The diversity of the TATA box and p53 binding sites in the third fragment (−263 to 0) is associated with the promoter activity of LTR5Hs.

**FIG 4:**
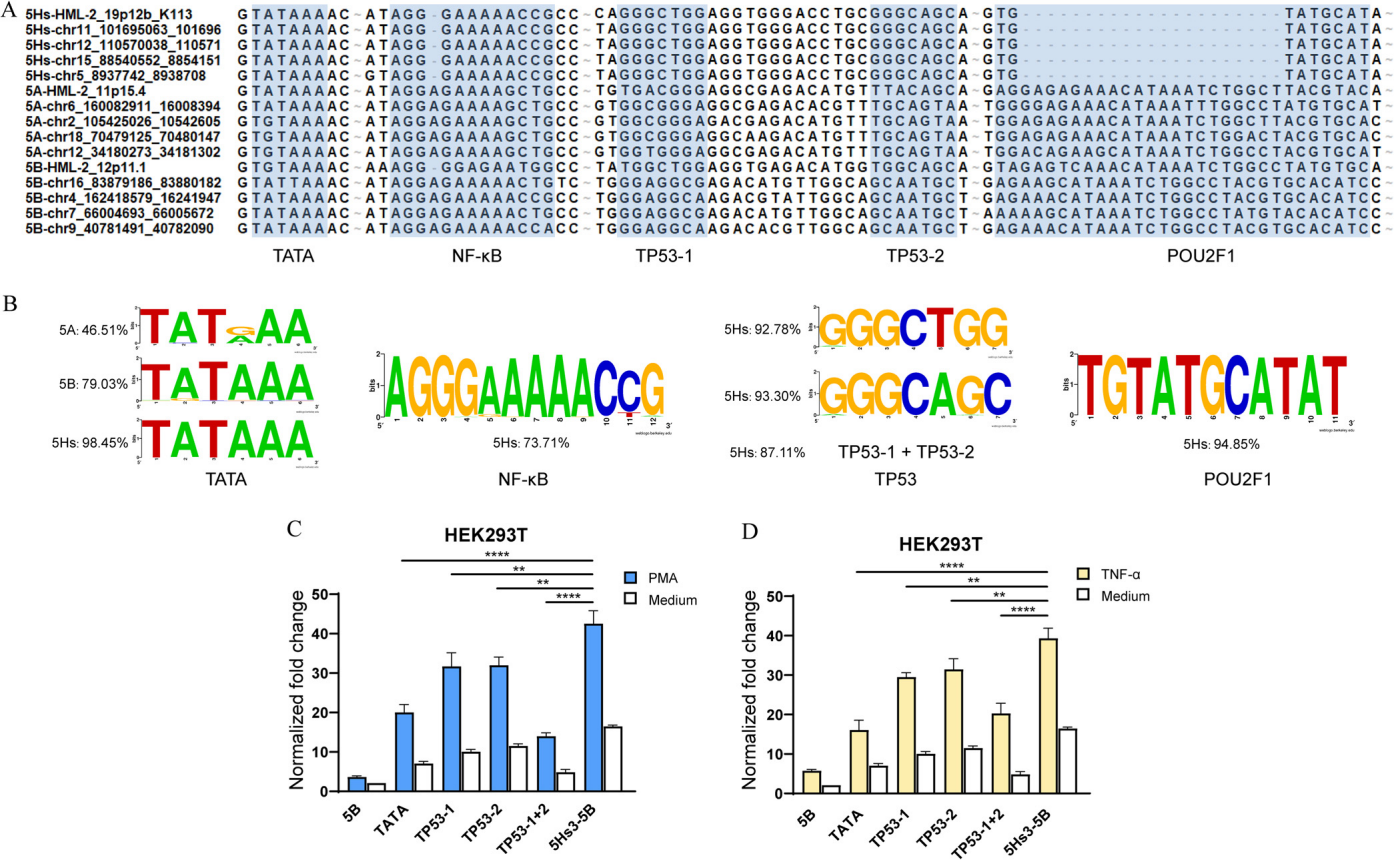
The TATA box and *TP53* TFBSs are associated with LTR5Hs activity. (A) Identification of the TFBSs in the −263-to-0 fragments of LTR5A, LTR5B, and LTR5Hs by Promo (http://alggen.lsi.upc.es/). (B) Conservation of the TATA motif in the 3 subtypes (http://weblogo.berkeley.edu/logo.cgi) and conservation of the *NF-κB*, *TP53*, and *POU2F1* motifs specifically in LTR5Hs. The frequency of each motif was determined using a total of 43 LTR5A elements, 62 LTR5B elements, and 194 LTR5Hs elements from the available reference sequences. (C and D) Promoter activities of constructs with different mutations. 5Hs3-5B luciferase reporter plasmids with mutations in TATA, *TP53-1*, *TP53-2*, or *TP53-1* and *TP53-2* binding sites with pRenilla-luc-TK were cotransfected into HEK293T cells for 24 h, and the cells were stimulated with PMA-ionomycin (C) or TNF-α (D) for an additional 24 h. The normalized fold change was obtained with the formula shown in Materials and Methods. Error bars indicate standard errors for the results of three independent experiments. **, *P* < 0.01; ****, *P* < 0.0001.

Based on the above-mentioned evidence, we next focused on the third fragments of the LTRs (nucleotides −263 to 0) to investigate how their diversity affected promoter activities using the dual-luciferase reporter system. This region contains multiple TFBSs. We analyzed the differences among the HML-2 LTR5A, LTR5B, and LTR5Hs subtypes and identified the following sites: the TATA box and the *NF-κB*, *TP53*, and *POU2F1* motifs (Fig. 4A). A total of 43 LTR5A sequences, 62 LTR5B sequences, and 194 LTR5Hs sequences were included in the statistical analysis. TATA boxes (TATAAAs) were found in 46.51%, 79.03%, and 98.45% of LTR5A, LTR5B, and LTR5Hs sequences, respectively. Other motifs conserved specifically in LTR5Hs included *NF-κB* (73.71%; AGGGAAAAACCG), *TP53-1* (92.78%; GGGCTGG), *TP53-2* (93.30%; GGGCAGC), *TP53-1* with *TP53-2* (87.11%), and *POU2F1* (94.85%; TGTATGCATAT) motifs (Fig. 4B).

To further study which TFBSs affect transcriptional activity, we mutated the above-mentioned TFBSs in LTR5Hs3-5B to the match corresponding loci of LTR5B. The constructs with mutations in the TATA box, *TP53-1*, and *TP53-2* binding sites of LTR5Hs3-5B exhibited significantly decreased transcriptional activity of this LTR. Importantly, the combination of the mutations in *TP53-1* and *TP53-2* decreased the transcriptional activity more significantly than either mutation alone (Fig. 4C and D). In addition, a luciferase assay was performed on the A549 and HepG2 cell lines. The results showed that the mutations in *TP53-1* and *TP53-2* binding sites in LTR5Hs3-5B significantly decreased the transcriptional activity of this LTR and that the combination of the mutations in *TP53-1* and *TP53-2* decreased the transcriptional activity more significantly than either mutation alone (Fig. S2A and B). In comparison, the mutations in either *NF-κB* or *POU2F1* sites did not significantly change the promoter activity compared with that of wild-type LTR5Hs3-5B in HEK293T cells (Fig. S2C).

### Silencing of *TP53* reduces LTR5Hs promoter activity.

**FIG 5:**
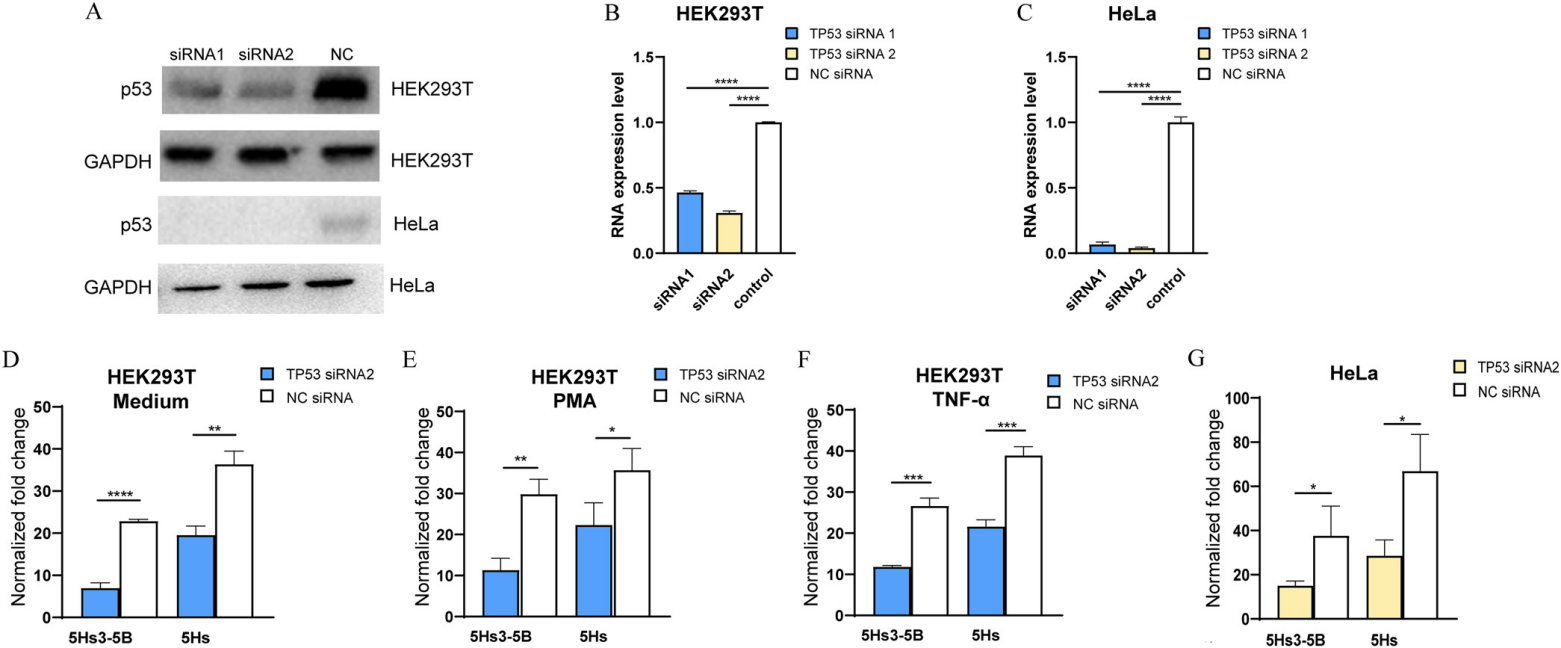
Inhibition of p53 reduces LTR5Hs promoter activity. (A) Immunoblot analysis of p53 after inhibition with siRNA in HEK293T and HeLa cells. Cells were transfected with a 50 nM concentration of either specific *TP53* siRNA1 or siRNA2 at 24 h posttransfection. GAPDH served as a loading control. The NC samples were treated with a random sequence. (B and C) qPCR of *TP53* RNA expression levels under two siRNAs. Total RNA treated with siRNA was isolated and amplified with primers specific for *TP53* in HEK293T (B) and HeLa (C) cell lines. The data are expressed as the fold increase in RNA over the level in NC cells. All qPCR results for each gene were normalized to those of the β-actin reference gene after analysis using the 2^−ΔΔ^*^CT^* method, and the relative expression is plotted. (D to G) Promoter activity of 5Hs3-5B and of LTR5Hs treated with *TP53* siRNA2. 5Hs3-5B or LTR5Hs luciferase reporter plasmids with pRenilla-luc-TK were cotransfected into HEK293T cells; cells were stimulated with *TP53* siRNA2 or a random sequence for 24 h and cultured in medium (D), PMA-ionomycin (E), or TNF-α (F) for an additional 24 h. 5Hs3-5B and LTR5Hs luciferase reporter plasmids with pRenilla-luc-TK were cotransfected into HeLa cells; cells were stimulated with *TP53* siRNA2 or a random sequence for 48 h (G). The normalized fold change was obtained with the formula in Materials and Methods. Error bars indicate standard errors for the results of three independent experiments. *, *P* < 0.05; **, *P* < 0.01; ***, *P* < 0.001; ****, *P* < 0.0001.

To elucidate the roles of *TP53* TFBSs in the promoter activity of LTR5Hs, we silenced the expression of the *TP53* with specific small interfering RNAs (siRNAs). Two siRNAs, siRNA1 and siRNA2, were designed to target the *TP53*. According to the results of Western blot analysis of p53 protein (Fig. 5A) and quantitative PCR (qPCR) of *TP53* RNA expression levels (Fig. 5B and C) in HEK293T and HeLa cells, siRNA2 showed a better interference effect and thus was selected for the follow-up experiments. First, we analyzed whether ablation of *TP53* by siRNA reduced LTR5Hs activity as determined by luciferase reporter assays. We observed that the promoter activity significantly decreased after silencing of *TP53* in both HEK293T cells (3.28-fold for LTR5Hs3-5B and 1.86-fold for LTR5Hs) and HeLa cells (2.5-fold for LTR5Hs3-5B and 2.34-fold for LTR5Hs) (Fig. 5D and G). Even different stimuli generated the same results in HEK293T cells. For LTR5Hs3-5B, there was 2.63-fold downregulation compared to the activity in the control group under PMA stimulation and 2.26-fold downregulation compared to the activity in the control group under TNF-α stimulation. For LTR5Hs, there was 1.60-fold downregulation compared to the activity in the control group under PMA stimulation and 1.80-fold downregulation compared to the activity in the control group under TNF-α stimulation (Fig. 5E and F). Together, these data demonstrate that p53 can target the LTR5Hs sequence to affect transcriptional activity.

### p53 directly regulates the promoter activity of LTR5Hs.

**FIG 6:**
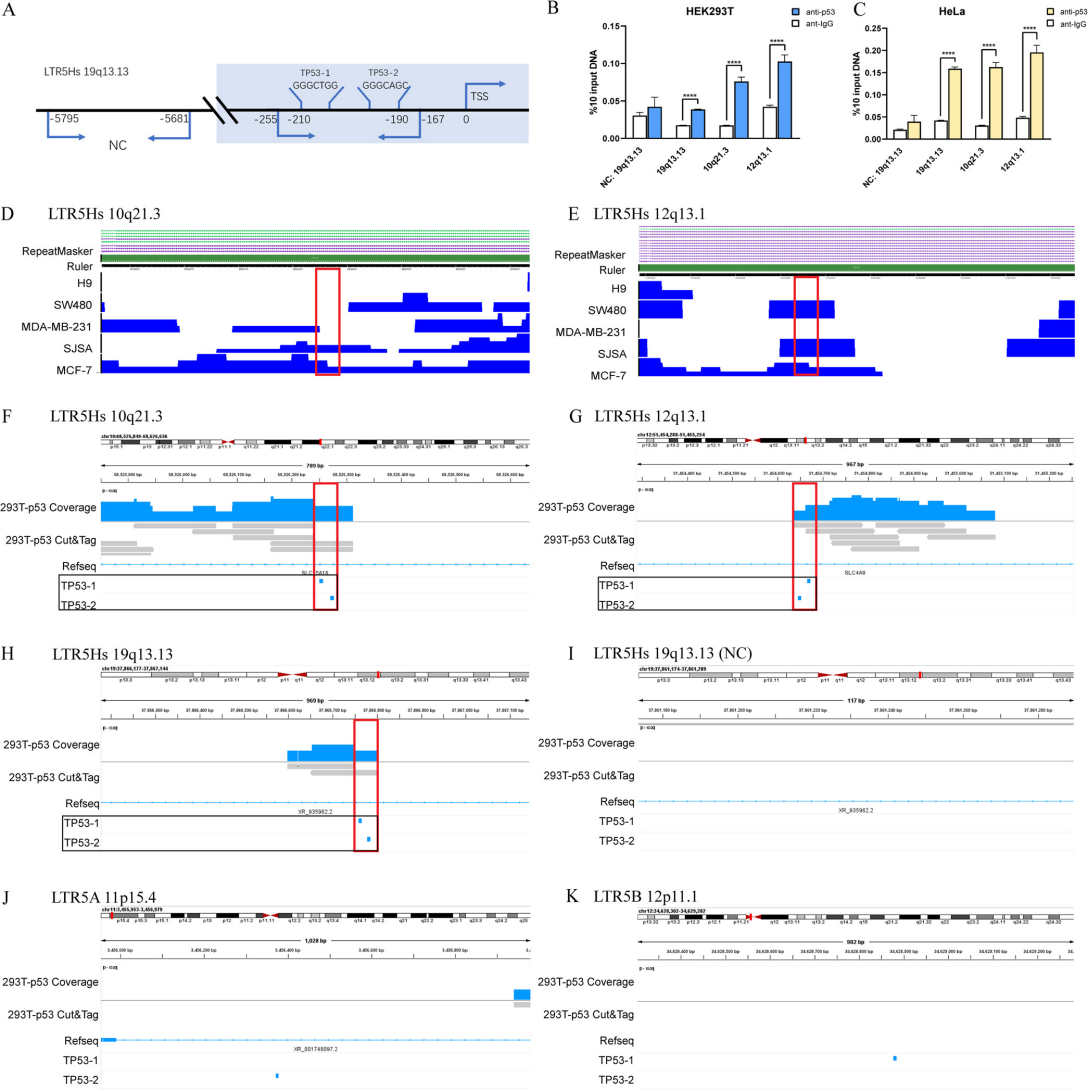
LTR5Hs elements are transactivated by p53. (A) Schematic illustration of the positions of the qPCR probes relative to the putative regions for ChIP-qPCR experiments. Chromatin pulled down by antibodies in HEK293T (B) and HeLa (C) cells was analyzed by qPCR. Error bars indicate standard errors of the means from three independent transfections. ****, *P* < 0.0001. (D and E) The p53 protein is enriched in other LTR5Hs elements according to the Cistrome Data Browser database (http://cistrome.org/db/#23/). The genomic loci include 10q21.3 (D) and 12q13.13 (E). The cell lines included H9 (human embryonic stem cells), SW480 (human colon cancer cells), MDA-MB-231 (human breast cancer cells), SJSA-1 (human osteosarcoma cells), and MCF-7 (human breast cancer cells). (F to K) p53 CUT&Tag profiling tracks of LTR5A, LTR5B, or LTR5Hs in different regions. CUT&Tag signals across ~1,000-bp genomic regions of LTR5Hs were selected over 10q21.3 (F), 12q13.13 (G), and 19q13.13 (H). The control regions, including the NC region of LTR5Hs (19q13.13) (I), LTR5A (11p15.4) (J), and LTR5B (12p11.1) (K), were visualized with IGV software. The red boxes represent the p53 binding region; the blue dot in the black boxes represent the *TP53-1* and *TP53-2* binding sites.

To verify the putative interaction between the LTR5Hs fragments and p53 protein, we performed chromatin immunoprecipitation (ChIP) followed by qPCR (ChIP-qPCR). The negative-control (NC) probe (19q13.13, chr19:37,861,174-37,861,289) was located 5 kb upstream from the transcription start site (TSS) of LTR5Hs, and the *TP53* probe encompassed both putative p53 binding regions (19q13.13, chr19:37,866,177-37,867,144) (Fig. 6A). In addition, we designed two other pairs of qPCR primers for p53 binding regions on LTR5Hs 10q21.3 (chr10:68,525,849-68,526,636) and 12q13.13 (chr12:51,454,288-51,455,254). The qPCR analysis of the chromatin pulled down by p53 antibodies suggested preferential p53 occupancy at the LTR5Hs sequence in HEK293T and HeLa cells (Fig. 6B and C), supporting the notion that p53 directly regulates LTR5Hs transcription. We also searched the Cistrome Data Browser database and found p53 enrichment at 10q21.3 and 12q13.13 of LTR5Hs. The cell lines included H9 (human embryonic stem cells), SW480 (human colon cancer cells), MDA-MB-231 (human breast cancer cells), SJSA-1 (human osteosarcoma cells), and MCF-7 (human breast cancer cells). The red boxes in Fig. 6 represent the loci of the p53 binding region. The ChIP followed by deep sequencing (ChIP-seq) results in the database showed that the p53 protein was enriched in 10q21.3 in SJSA-1 and MCF-7 cells and enriched in 12q13.13 in SW480, SJSA-1, and MCF-7 cells (Fig. 6D and E).

To further confirm the binding regions between LTR5Hs and the p53 protein, we performed a CUT&Tag (cleavage under targets and tagmentation) assay. The p53 CUT&Tag profiling tracks of LTR5Hs are displayed over chromosomes, including 10q21.3, 12q13.13, and 19q13.13 (Fig. 6F to H). The high resolution of CUT&Tag provided structural details for individual sites. The superposition of p53 mapping at the representative sites revealed the binding regions between the p53 protein and LTR5Hs. In Fig. 6, the red boxes represent the p53 binding region, and the blue dot in the black boxes represent the *TP53-1* and *TP53-2* binding sites. Notably, there were no peaks in the NC (19q13.13), LTR5A (11p15.4), or LTR5B (12p11.1), as shown in Fig. 6I to K. In general, the results of the experiment confirmed our inference that these two p53 binding sites of LTR5Hs may be responsible for the differences in transcriptional activity between LTR5Hs and LTR5A/5B.

We further verified the other two loci, 7q22.1 (chr7:101,149,855-101,150,821) and 11q13.2 (chr11:67,603,354-67,604,197), using the same types of experiments. The results of ChIP-qPCR of the chromatin pulled down by p53 antibodies in HEK293T and HeLa cells are shown in Fig. S3A and B. The Cistrome Data Browser data on p53 enrichment at 7q22.1 and 11q13.2 are shown in Fig. S3C and D. The red boxes represent the p53 binding region. The ChIP-seq results on database showed that the p53 protein was enriched in 7q22.1 in H9 and MCF-7 cells and enriched in 11q13.2 in MCF-7 cells. The p53 CUT&Tag profiling tracks of LTR5Hs are displayed over chromosomes, including 7q22.1 and 11q13.2, as shown in Fig. S3E and F. The superposition of p53 mapping at the representative sites revealed the binding region between the p53 protein and LTR5Hs.

Additionally, based on the CUT&Tag profiling track results, we did not find a p53 binding site in the homologous fragment (−263 to 0) or p53 enrichment in the 43 LTR5A and 62 LTR5B sequences, which was consistent with the BLAT results in Human Genome Assembly 38 (hg38). Among the 194 LTR5Hs sequences, 169 of these (87.11%) had *TP53-1* and *TP53-2* binding sites in nucleotides −263 to 0. Among these 169 sequences, 72 of these (42.60%) were identified as being enriched with the two p53 binding sites.

## DISCUSSION

HERV LTRs are often categorized as transposable elements and harbor principal core promoter elements as well as important transcriptional enhancers. Indeed, there are many examples of HERV-K (HML-2) LTRs that feature novel gene promoters or TFBSs (26, 27).

HERV-K (HML-2) LTR5Hs contains two conserved interferon-stimulation response elements (ISREs; GGAAGGGAAA and GATAGGAAA) that overlap active and intact *NF-κB* and *NFAT-1* binding sites. The nuclear translocation of *IRF1* and *NF-κB* results in upregulation of HERV-K in ALS (28).

*YY1* (CCATNTT), a ubiquitous transcription factor that binds to the 5′ termini of the U3 regions of HERV-K (HML-2) LTR5A, LTR5B, and LTR5Hs, has been shown to activate HERV-K expression in many cell lines, including HepG2, HeLa, GH, and Tera2 cells. Mutation of this *YY1* binding site causes a 50% reduction in the activity of the HERV-K LTR (3, 29).

Melanoma-specific microphthalmia-associated transcription factor (*MITF-M*) is an oncogene in melanoma. *MITF-M* (CACATG, CTTGTG, CACATC, and CATATG) activates the responsive sequences (E boxes) in the partial sequences of HERV-K (HML-2) LTR5A, LTR5B, and LTR5Hs, leading to high-level expression in malignant melanoma (30).

The splicing factor RNA-binding motif 4 (*RBM4*) suppresses the proliferation and migration of various cancer cells by specifically controlling cancer-related splicing. Its reduced expression is related to poor overall survival in lung, breast, ovarian, and gastric cancers (31). *RBM4* has been identified to be negatively correlated with the expression of HERV-K (HML-2) and to bind HML-2 LTR5A, LTR5B, and LTR5Hs at CGG consensus elements. *RBM4* knockout leads to HML-2 transcript upregulation and increases Env protein expression in the chronic myelogenous leukemia (CML) cell line HAP1 (32).

LTRs can alter the regulation of nearby genes and potentially influence the control of genes thousands of base pairs away (33–35). In our study, we focused on HML-2, the most biologically active subgroup of HERV-K. HML-2 LTR sequences can be classified into the LTR5A, LTR5B, and LTR5Hs subtypes. Some studies have confirmed that these three subtypes regulate different genes. Activation/silencing of LTR5Hs has been suggested to be associated with reciprocal up- and downregulation of hundreds of human genes (33). The *OCT4* motif (ATGCAAA/ATGCCAA) at base pairs 693 to 699 of LTR5Hs is conserved across diverse LTR5Hs sequences but is not present in LTR5A or LTR5B. This binding site on HML-2 LTR5Hs can promote HERV-K transcription in HEK293T cells (36).

Based on the research progress thus far, we attempted to identify the mechanisms underlying the differences in LTR transcriptional activity among the three subtypes.

First, we characterized HML-2 LTRs in hg38 into three subgroups: LTR5A, LTR5B, and LTR5Hs. Although some previous studies performed similar work, our BLAT results were based on the Dfam-derived consensus sequence combined with literature-derived reference sequences, which made our sequence identification more accurate and comprehensive. The phylogenetic analysis showed that LTR5Hs was clearly separated from the other 2 subtypes, whereas LTR5A sequences were nested within the LTR5B sequences. Our tree topology is consistent with previous reports (13, 17, 24, 25). The results support the theory that the LTR5B subgroup is the oldest and most ancestral, while LTR5A and LTR5Hs independently originated from the LTR5B group. However, as LTR5A sequences were nested within the LTR5B sequences, LTR5A should not be a distinct cluster; rather, it should be assigned to the LTR5B cluster. Thus, there is a need to reclassify the LTR subgroups.

Next, we conducted a predictive analysis of regulated genes and then performed GO analysis. Nine potential regulatory genes were shared by the three subtypes, while LTR5A and LTR5B shared 99 regulatory genes. In addition, the genes that can be regulated by LTR5A, LTR5B, and LTR5Hs had almost completely distinct BP and MF terms. These results are entirely speculative; thus, additional research is needed.

Based on the above-mentioned bioinformatics analysis results, we selected representative sequences of the three subtypes to construct the luciferase-based reporter vector pGL4.17. The luciferase reporter assay results showed that LTR5Hs had a stronger promoter activity than LTR5A/LTR5B in the HEK293T, HeLa, Beas-2B, L02, A549, HepG2, MT-2, and Jurkat T cell lines. We successively replaced 4 regions of LTR5B with their homologous fragments in LTR5Hs and found that the third section (−263 to 0) of LTR5Hs significantly increased LTR5B promoter activity. The results suggested that the fragment from −263 to 0 containing the enhancer/core promoter sequence determines the difference in LTR activity of LTR5Hs.

We focused further on the third section and screened 5 different TFBSs: the TATA box, *NF-κB*, *TP53-1*, *TP53-2*, and *POU2F1* binding sites. As these elements were replaced by homologous fragments of LTR5B, the luciferase reporter assay results showed that the TATA box, *TP53-1*, and *TP53-2* binding sites specifically influenced the promoter activity of the third section (LTR5Hs3-5B) of LTR5Hs. TATA-binding protein (TBP) is a transcription factor that specifically binds to the TATA box. For this TFBS, the TATAA variant TGTAA may affect the binding efficiency of the protein. This situation is similar to that in human immunodeficiency virus (HIV): the subtype CRF01_AE contains a different TBP binding site (with the sequence TAAAA), whereas all other subtypes have the TATAA sequence. This single-nucleotide variation reduces the assembly efficiency of the TBP-TFIIB-TATA complex for the recruitment of RNA polymerase II (37). Considering that the TATA box was not present only in the LTR5Hs subtype (TATAAA was present in 46.51% and 79.03% of LTR5A and LTR5B sequences, respectively), we did not conduct in-depth research on its mechanism.

p53, an important tumor suppressor protein, is a specific transcription factor that regulates the expression of genes related to cell cycle arrest, organic processes, and apoptosis in response to cellular stress and is critical for the control of homeostasis (38–40). Previous ChIP and expression studies on individual genes have suggested that p53 sites in HERV LTRs may be part of the p53 transcriptional program and directly regulate p53 target genes in a species-specific manner (6, 41). However, the exact function of p53 in the regulation of HERV LTR expression has not been studied.

In the current work, we focused on the 3 LTR subtypes of HERV-K (HML-2), which is the most biologically active HERV subgroup, and directly revealed that the two unique p53 binding sites located in the −263 to 0 region of the LTR5Hs subtype pose crucial roles to promote LTR5Hs transcriptional activity. The activity of LTR5Hs was decreased by silencing of *TP53*. Furthermore, the ChIP-qPCR assay and CUT&Tag assay proved the direct binding of the p53 protein with LTR5Hs sequences. Interestingly, CUT&Tag showed that there was no direct binding of p53 with LTR5A (11p15.4) or LTR5B (12p11.1) sequences, making up for the shortcomings of ChIP-qPCR. One advantage of CUT&Tag is that the entire reaction from antibody binding to adaptor integration occurs within intact cells. Antibodies are easier to detect than with other approaches, which makes the CUT&Tag method more sensitive (42, 43). We suspect that ChIP-qPCR could not distinguish the enrichment of LTR5A/LTR5B because of the adjacent p53 binding sites on LTR5B. Additionally, based on the CUT&Tag profiling tracks, there were 169 sequences (87.11%) with *TP53-1* and *TP53-2* motifs in the region from −263 to 0 among the 194 LTR5Hs loci. Among these 169 sequences, 72 (42.60%) were identified to be enriched with the two p53 binding sites.

*TP53* mutation has profound effects on tumor cell genomic structure, gene expression, and clinical outlook. Mutations in *TP53* most commonly occur as single-nucleotide variants at positions that cluster in the DNA-binding domain (44, 45). Rs28934578 (R175H), in which arginine at position 175 is replaced with histidine, is the most common mutation observed in *TP53* in many tumor types (46). This *TP53* hot spot mutation was located in the locus of our p53 binding site (GCTGCCC). Further analysis of The Cancer Genome Atlas (TCGA) database revealed that there were 5 single-nucleotide polymorphism (SNP) sites located in p53 binding sites, including rs587783062, rs587781504, rs28934578, rs786202962, and rs147002414. The cancer types and SNP loci and proportions are shown in Table S5.

Taken together, our findings characterize the promoter activity differences among LTR5A, LTR5B, and LTR5Hs and reveal the critical role of the two p53 binding sites in LTR5Hs. p53 has a critical tumor suppressor function. Therefore, our findings suggest that the most integrated and most active LTR of HERV-K (HML-2), LTR5Hs, probably participates in the tumor suppressor function of p53. Notably, more than 30% of all p53 binding sites in the whole human genome are located in HERV LTRs (41). Thus, our findings are critical to understanding the roles of HERV LTRs in the regulatory process and may provide a basis for future comprehensive study of the physiological and pathological functions.

## MATERIALS AND METHODS

### Reference sequence selection.

In order to prevent bias and optimize representativeness, we selected the reference sequences in this study from two sources. One source was the group of consensus representatives from the Dfam database (https://www.dfam.org/home), including LTR5A_DF0000556.4, LTR5B_DF0000557.4 and LTR5Hs_DF0000558.4 (47). The other source was reference 2, which provided references including LTR5A_11p15.4, LTR5B_12p11.1, and LTR5Hs_19p12b_K113 (2). The similarity between LTR5A_DF0000556.4 and LTR5A_11p15.4 was 93.4%, the similarity between LTR5B_DF0000557.4 and LTR5B_12p11.1 was 82.3%, and the similarity between LTR5Hs_DF0000558.4 and LTR5Hs_19p12b_K113 was 96.6%. The Dfam-derived consensus representatives were used to perform BLAT-based element identification. The reference 2-derived representatives were used to construct the targeted plasmid vectors and perform experimental tests. Both representative groups were used to construct phylogenetic trees.

We further compared each representative sequence with all other elements of its subgroup. After gap stripping, the similarity between LTR5A_DF0000556.4_LTR5A and the other 43 LTR5A elements was 94.21% ± 1.11%, the similarity between LTR5A_11p15.4 and the other 43 LTR5A elements was 91.23% ± 2.06%, the similarity between DF0000557.4_LTR5B and the other 62 LTR5B elements was 91.16% ± 1.41%, the similarity between LTR5B_12p11.1 and the other 62 LTR5B elements was 79.13% ± 1.35%, the similarity between DF0000558.4_LTR5Hs and the other 194 LTR5Hs elements was 98.25% ± 0.68%, and the similarity between LTR5Hs_19p12b_K113 and the other 194 LTR5Hs elements was 95.75% ± 1.30%. LTR5B is the oldest ancestral type and has thus experienced the longest period of evolution, while LTR5Hs is the most recently acquired type (5). Since it is the newest LTR, it has had the least time to accumulate mutations leading to inactivation. Our data also show the order of similarity of LTR5B, LTR5A, and LTR5Hs from low to high (Table S6). Given the strong validity of our approach, the representative strains we selected can also be used for other members of the same subgroup.

### *In silico* identification of HML-2 sequences in hg38.

The Genome Reference Consortium December 2013 release of the assembly hg38 was used as the human reference sequence to perform BLAT-based identification of the chromosomal coordinates of HML-2 elements. Three HML-2 LTR subgroups in Dfam (http://dfam.org), DF0000556.4_LTR5A, DF0000557.4_LTR5B, and DF0000558.4_LTR5Hs, were used as representatives (47). The BLAT search on the Ensembl website (https://asia.ensembl.org/index.html) was performed on hg38 using the Dfam-derived HML-2 LTR5A, LTR5B, and LTR5Hs references (48). DNA BLAT creates an index of the entire genome that consists of all overlapping 11-mers in steps of 5 except for those heavily involved in repeats. The identified sequences were extracted in FASTA format with TBtools (49). All identified elements were aligned using ClustalW and manually edited in BioEdit v.7.2.5 (50). Sequences longer than 60% of the Dfam-derived full-length representatives of each subtype were selected for further analyses.

### Phylogenetic analyses.

ML phylogenetic trees were constructed to confirm the assignment of BLAT-derived HML-2 LTR sequences using Mega X (51). LTR5A_11p15.4, LTR5B_12p11.1, and LTR5Hs_19p12b_K113, which were used as reference sequences in a previous study (2), and the Dfam-derived sequences mentioned above were used as the reference sequences for the 3 types of LTRs. The base model selection function of Mega X was used, and the best-fitting model of nucleotide substitution for LTR analysis was K2+G. The tree topologies were searched using the nearest-neighbor interchange (NNI) procedure. The confidence of each node in phylogenetic trees was determined using the bootstrap test with 1,000 bootstrap replicates. The final ML trees were visualized using the web tool iTOL (52).

### Prediction of the regulatory functions of LTRs.

To predict the regulatory roles of LTR5A, LTR5B, and LTR5Hs in the human genome, the nearby genes were annotated with the tool GREAT (53). The association rule was as follows: basal + extension: 5,000 bp upstream, 1,000 bp downstream, 1,000,000 bp maximum extension, curated regulatory domains included. Then, we used Bioinformatics & Evolutionary Genomics, an online tool (http://bioinformatics.psb.ugent.be/webtools/Venn), to create custom Venn diagrams for the potential genes regulated by the three subtypes.

### GO enrichment analyses.

The Database for Annotation, Visualization and Integrated Discovery (DAVID, http://david.ncifcrf.gov; version 6.8) is an online biological information database that integrates biological data and analysis tools and provides a comprehensive set of functional annotation information for genes and proteins, from which users can extract biological information (54). GO is a major bioinformatics tool to annotate genes and analyze the BPs and MFs of these genes (55). Biological analyses were performed using DAVID. *P* values of <0.05 were considered to indicate statistical significance. The results were visualized with a Sankey diagram generated with the R package ggalluvial.

### Cell culture.

All cell lines were obtained from the American Type Culture Collection (ATCC). HEK293T, HeLa, A549, and HepG2 cells were grown in Dulbecco’s modified Eagle’s medium (DMEM; Thermo Fisher Scientific, Waltham, MA, USA); Beas-2B cells were grown in minimum essential medium (MEM; Thermo Fisher Scientific); and L02, MT-2, and Jurkat T cells were cultured in RPMI 1640 medium (Thermo Fisher Scientific) supplemented with 10% fetal bovine serum (FBS) (Thermo Fisher Scientific) and 1% penicillin-streptomycin (Thermo Fisher Scientific). All cell lines were maintained at 37°C in a humidified incubator containing 5% CO_2_ in air.

### Plasmid constructs.

The sequences and reverse sequences of HML-2 LTR5A (11p15.4, chr11:3,455,953-3,456,979), HML-2 LTR5B (12p11.1, chr12:34,628,302-34,629,282), and HML-2 LTR5Hs (19p12b_K113, chr19:37,866,177-37,867,144) were cloned into the KpnI and NheI restriction sites of pGL4.17 (Promega, Madison, WI, USA) to perform luciferase reporter assays. The pRenilla-luc-TK luciferase plasmid pGL4.74 was obtained from Promega. LTR5Hs was divided into four sections, which were used to replace the corresponding positions of LTR5B with a seamless cloning kit (Beyotime, Shanghai, China). All primer sequences are given in the 5′-to-3′ direction. The primers used to construct the four sections of LTR5Hs to generate the LTR5B luciferase reporters (5Hs1-5B-Luc, 5Hs2-5B-Luc, 5Hs3-5B-Luc, and 5Hs4-5B-Luc) are shown in Table S7.

The TATA box, *NF-κB*, *TP53-1*, *TP53-2*, and *POU2F1* binding sites in LTR5Hs3-5B-Luc vector were mutated to the corresponding sequences of LTR5B with a Q5 site-directed mutagenesis kit (New England Biolabs [NEB], MA, USA). The TATA box was mutated from 5′-TATAA-3′ to 5′-TGTAA-3′, the *NF-κB* binding site was mutated from 5′-AGGGAAAAACCG-3′ to 5′-AGGGGAGAATGG-3′, the *TP53-1* binding site was mutated from 5′-GGGCTGG-3′ to 5′-TGGCTGG-3′, the *TP53-2* binding site was mutated from 5′-GGGCAGC-3′ to 5′-TGGCAGC-3′, and the *POU2F1* binding site was mutated from 5′-TGTATGCATAT-3′ to 5′-TAGAGTCAAACATAAATCTGGCCTATGTGC-3′. The primers used to create the mutant constructs are shown in Table S7.

### Dual-luciferase reporter assays.

HEK293T, HeLa, Beas-2B, L02, A549, and HepG2 cells were seeded in 96-well cell culture plates for 24 h before transfection. Transfection was performed using Lipofectamine 2000 (Invitrogen) according to the manufacturer’s instructions. All transfections included *Renilla* luciferase as an internal control (pRenilla-luc-TK) to assess for variation in transfection efficiency. The amounts of plasmids used for transfections in 96-well cell culture plates were as follows: 200 ng of luciferase reporter plasmid (HERV-K [HML-2] LTR) or empty luciferase vector pGL4.17, with 5 ng pRenilla-luc-TK. At 6 h posttransfection, the medium containing a mixture of plasmids and transfection reagent was replaced with fresh medium supplemented with 10% FBS. At 24 h posttransfection, the cells were treated with TNF-α (50 ng/mL) or PMA (20 nM)/ionomycin (1.5 μM). At 48 h posttransfection, the transfected cells were assayed for luciferase activity using a dual-luciferase assay kit (Promega) according to the manufacturer’s instructions.

To display the difference of promoter activity between different constructs in the same stimulation, as well as the difference of promoter activity of the same construct in different stimulation (cell culture medium and PMA-ionomycin or TNF-α), the normalized fold change were calculated with the following formula: (average [firefly luminescence/*Renilla* luminescence] experimental luciferase units in a specific stimulus)/(average [firefly luminescence/*Renilla* luminescence] experimental luciferase units using empty pGL4.17 in cell culture medium rather than in the same stimulus). The experiment under each condition was performed with at least three independent biological replicates.

### Cell transfection with siRNA oligonucleotides.

*TP53* mRNA was silenced with 50 nM specific *TP53* siRNA (Sangon, Shanghai, China) by using Lipofectamine 2000 (Invitrogen). The NC siRNA was a random sequence (50 nM). At 24 h after transfection, the treated cells were harvested for Western blot analysis and real-time RT-PCR. The siRNA sequences are shown in Table S7.

### RNA extraction and real-time RT-PCR.

Total cellular RNA was isolated from cells using a MiniBEST universal RNA extraction kit (TaKaRa). The RNA concentration was measured with a NanoDrop spectrophotometer. The acceptable RNA purity was an *A*_260_/*A*_280_ value of 1.95 to 2.05. A PrimeScript RT reagent kit with gDNA Eraser (TaKaRa) was used to synthesize cDNA from the extracted RNA according to the manufacturer’s instructions.

qPCR was performed using the SYBR green detection method (TB green premix Ex Taq; TaKaRa) in a LightCycler 480 (Roche, Basel, Switzerland) to measure *TP53* RNA expression levels after silencing with siRNA. The primers used are shown in Table S7. qPCR was performed on technical duplicates or triplicates of each sample. Each condition was performed with three independent biological replicates.

### Protein extraction and Western blotting.

Cells were washed with ice-cold phosphate-buffered saline (PBS) twice and then incubated with lysis buffer on ice for 20 min. The cell lysates were centrifuged at 12,000 × *g* for 15 min at 4°C. The protein concentrations were measured by the bicinchoninic acid (BCA) (TaKaRa) method to determine the protein content of each sample. The cell lysates were prepared for sodium dodecyl sulfate (SDS)-polyacrylamide gel electrophoresis (PAGE), heated at 95°C for 10 min, and then transferred to polyvinylidene difluoride (PVDF) membranes. The membranes were blocked in a 5% skim milk solution for 1 h and then probed with primary antibodies against p53 (1:1,000 dilution) and GAPDH (1:20,000 dilution) overnight at 4°C. After washing with PBS with Tween 20 (PBST) for 1 h, the membranes were incubated with secondary antibodies (1:3,000 dilution). Then, the membranes were imaged using chemiluminescent horseradish peroxidase (HRP) substrate (Millipore, Billerica, MA, USA) after washing with PBST for 1 h.

The primary antibodies and HRP-conjugated secondary antibodies used were as follows: anti-p53 (10442-1-AP; Proteintech, Wuhan, China) and anti-GAPDH (60004-1-Ig; Proteintech) primary antibodies and goat anti-mouse or anti-rabbit IgG secondary antibodies (SA00001-1 and SA00001-2; Proteintech).

### ChIP-qPCR.

ChIP was performed with a ChIP kit (ab500; Abcam, Cambridge, UK) according to the manufacturer’s instructions. Approximately 3 × 10^6^ cells were harvested for ChIP. Briefly, the cells were cross-linked with 1% formaldehyde for 10 min at room temperature. Glycine was added to stop the cross-linking reaction, and the cells were washed with ice-cold PBS. Following solution removal, the tubes were chilled on ice, and the cells were lysed using ice-cold cell lysis buffer containing protease inhibitors and phenylmethylsulfonyl fluoride (PMSF). The chromatin was fragmented to 200 to 500 bp with a Bioruptor Plus (Diagenode, Belgium) at 4°C. After centrifugation, the chromatin supernatants were diluted with cold dilution buffer. Subsequently, a human p53 antibody (4 μg; 10442-1-AP) was added to the chromatin, and the mixture was incubated at 4°C overnight. The antibody-chromatin samples were pelleted and incubated with protein A beads. After incubation, the antibody-chromatin/beads were washed 4 times, and DNA was purified with the DNA-purifying slurry included in the ChIP kit. Finally, the purified DNA was used for qPCR analysis using the SYBR green detection method. The ChIP-qPCR primers used are shown in Table S7.

### CUT&Tag library generation and sequencing.

CUT&Tag was performed with a Hyperactive *in situ* ChIP library preparation kit for Illumina (Vazyme Biotech; TD901). In brief, the cells were incubated with 10 μL of prewashed ConA beads in a low-binding tube. Then, we added 50 μL of antibody buffer with 0.5 μg of antibody and cultured the cells for 2 h at room temperature. After washing twice, 50 μL of digitonin (Dig) wash buffer with 0.5 μg of secondary antibody was added, and the samples were incubated at room temperature for 30 min. After washing twice, we added 0.58 μL of Hyperactive pG-Tn5/pA-Tn5 transposon with 100 μL of Dig-wash buffer and incubated the samples at room temperature for 1 h. After washing twice, 300 μL of tagmentation buffer was added, and the samples were incubated at 37°C for 1 h. After DNA extraction with the phenol-chloroform and ethanol precipitation method, PCR was performed to amplify the libraries. All libraries were sequenced with an Illumina NovaSeq 6000 instrument. The CUT&Tag data were visualized with Integrative Genomics Viewer (IGV) software (56).

## References

[1] Bock M, Stoye JP. 2000. Endogenous retroviruses and the human germline. Curr Opin Genet Dev 10:651–655. doi:10.1016/s0959-437x(00)00138-6. 11088016

[2] Subramanian RP, Wildschutte JH, Russo C, Coffin JM. 2011. Identification, characterization, and comparative genomic distribution of the HERV-K (HML-2) group of human endogenous retroviruses. Retrovirology 8:90. doi:10.1186/1742-4690-8-90. 22067224 PMC3228705

[3] Manghera M, Douville RN. 2013. Endogenous retrovirus-K promoter: a landing strip for inflammatory transcription factors? Retrovirology 10:16. doi:10.1186/1742-4690-10-16. 23394165 PMC3598470

[4] Medstrand P, van de Lagemaat LN, Mager DL. 2002. Retroelement distributions in the human genome: variations associated with age and proximity to genes. Genome Res 12:1483–1495. doi:10.1101/gr.388902. 12368240 PMC187529

[5] Xue B, Sechi LA, Kelvin DJ. 2020. Human endogenous retrovirus K (HML-2) in health and disease. Front Microbiol 11:1690. doi:10.3389/fmicb.2020.01690. 32765477 PMC7380069

[6] Garcia-Montojo M, Doucet-O’Hare T, Henderson L, Nath A. 2018. Human endogenous retrovirus-K (HML-2): a comprehensive review. Crit Rev Microbiol 44:715–738. doi:10.1080/1040841X.2018.1501345. 30318978 PMC6342650

[7] Wallace TA, Downey RF, Seufert CJ, Schetter A, Dorsey TH, Johnson CA, Goldman R, Loffredo CA, Yan P, Sullivan FJ, Giles FJ, Wang-Johanning F, Ambs S, Glynn SA. 2014. Elevated HERV-K mRNA expression in PBMC is associated with a prostate cancer diagnosis particularly in older men and smokers. Carcinogenesis 35:2074–2083. doi:10.1093/carcin/bgu114. 24858205 PMC4146419

[8] Vargiu L, Rodriguez-Tomé P, Sperber GO, Cadeddu M, Grandi N, Blikstad V, Tramontano E, Blomberg J. 2016. Classification and characterization of human endogenous retroviruses; mosaic forms are common. Retrovirology 13:7. doi:10.1186/s12977-015-0232-y. 26800882 PMC4724089

[9] Arru G, Galleri G, Deiana GA, Zarbo IR, Sechi E, Bo M, Cadoni MPL, Corda DG, Frau C, Simula ER, Manca MA, Galistu F, Solla P, Manetti R, Sechi GP, Sechi LA. 2021. HERV-K modulates the immune response in ALS patients. Microorganisms 9:1784. doi:10.3390/microorganisms9081784. 34442863 PMC8399181

[10] Mameli G, Erre GL, Caggiu E, Mura S, Cossu D, Bo M, Cadoni ML, Piras A, Mundula N, Colombo E, Buscetta G, Passiu G, Sechi LA. 2017. Identification of a HERV-K env surface peptide highly recognized in rheumatoid arthritis (RA) patients: a cross-sectional case-control study. Clin Exp Immunol 189:127–131. doi:10.1111/cei.12964. 28324619 PMC5461087

[11] Arru G, Mameli G, Deiana GA, Rassu AL, Piredda R, Sechi E, Caggiu E, Bo M, Nako E, Urso D, Mariotto S, Ferrari S, Zanusso G, Monaco S, Sechi G, Sechi LA. 2018. Humoral immunity response to human endogenous retroviruses K/W differentiates between amyotrophic lateral sclerosis and other neurological diseases. Eur J Neurol 25:1076–1e84. doi:10.1111/ene.13648. 29603839

[12] Hohn O, Hanke K, Bannert N. 2013. HERV-K(HML-2), the best preserved family of HERVs: endogenization, expression, and implications in health and disease. Front Oncol 3:246. doi:10.3389/fonc.2013.00246. 24066280 PMC3778440

[13] Buzdin A, Ustyugova S, Khodosevich K, Mamedov I, Lebedev Y, Hunsmann G, Sverdlov E. 2003. Human-specific subfamilies of HERV-K (HML-2) long terminal repeats: three master genes were active simultaneously during branching of hominoid lineages. Genomics 81:149–156. doi:10.1016/s0888-7543(02)00027-7. 12620392

[14] Dunn CA, van de Lagemaat LN, Baillie GJ, Mager DL. 2005. Endogenous retrovirus long terminal repeats as ready-to-use mobile promoters: the case of primate beta3GAL-T5. Gene 364:2–12. doi:10.1016/j.gene.2005.05.045. 16112824

[15] Ehlhardt S, Seifert M, Schneider J, Ojak A, Zang KD, Mehraein Y. 2006. Human endogenous retrovirus HERV-K(HML-2) Rec expression and transcriptional activities in normal and rheumatoid arthritis synovia. J Rheumatol 33:16–23. 16395745

[16] Ito J, Sugimoto R, Nakaoka H, Yamada S, Kimura T, Hayano T, Inoue I. 2017. Systematic identification and characterization of regulatory elements derived from human endogenous retroviruses. PLoS Genet 13:e1006883. doi:10.1371/journal.pgen.1006883. 28700586 PMC5529029

[17] Macfarlane C, Simmonds P. 2004. Allelic variation of HERV-K(HML-2) endogenous retroviral elements in human populations. J Mol Evol 59:642–656. doi:10.1007/s00239-004-2656-1. 15693620

[18] Hughes JF, Coffin JM. 2004. Human endogenous retrovirus K solo-LTR formation and insertional polymorphisms: implications for human and viral evolution. Proc Natl Acad Sci USA 101:1668–1672. doi:10.1073/pnas.0307885100. 14757818 PMC341815

[19] Buzdin A, Kovalskaya-Alexandrova E, Gogvadze E, Sverdlov E. 2006. At least 50% of human-specific HERV-K (HML-2) long terminal repeats serve in vivo as active promoters for host nonrepetitive DNA transcription. J Virol 80:10752–10762. doi:10.1128/JVI.00871-06. 17041225 PMC1641792

[20] Yu HL, Zhao ZK, Zhu F. 2013. The role of human endogenous retroviral long terminal repeat sequences in human cancer (review). Int J Mol Med 32:755–762. doi:10.3892/ijmm.2013.1460. 23900638

[21] Smit AF. 1999. Interspersed repeats and other mementos of transposable elements in mammalian genomes. Curr Opin Genet Dev 9:657–663. doi:10.1016/s0959-437x(99)00031-3. 10607616

[22] Cohen CJ, Lock WM, Mager DL. 2009. Endogenous retroviral LTRs as promoters for human genes: a critical assessment. Gene 448:105–114. doi:10.1016/j.gene.2009.06.020. 19577618

[23] Xue B, Zeng T, Jia L, Yang D, Lin SL, Sechi LA, Kelvin DJ. 2020. Identification of the distribution of human endogenous retroviruses K (HML-2) by PCR-based target enrichment sequencing. Retrovirology 17:10. doi:10.1186/s12977-020-00519-z. 32375827 PMC7201656

[24] Belshaw R, Pereira V, Katzourakis A, Talbot G, Paces J, Burt A, Tristem M. 2004. Long-term reinfection of the human genome by endogenous retroviruses. Proc Natl Acad Sci USA 101:4894–4899. doi:10.1073/pnas.0307800101. 15044706 PMC387345

[25] Hughes JF, Coffin JM. 2001. Evidence for genomic rearrangements mediated by human endogenous retroviruses during primate evolution. Nat Genet 29:487–489. doi:10.1038/ng775. 11704760

[26] Kapusta A, Kronenberg Z, Lynch VJ, Zhuo X, Ramsay L, Bourque G, Yandell M, Feschotte C. 2013. Transposable elements are major contributors to the origin, diversification, and regulation of vertebrate long noncoding RNAs. PLoS Genet 9:e1003470. doi:10.1371/journal.pgen.1003470. 23637635 PMC3636048

[27] Fort A, Hashimoto K, Yamada D, Salimullah M, Keya CA, Saxena A, Bonetti A, Voineagu I, Bertin N, Kratz A, Noro Y, Wong CH, de Hoon M, Andersson R, Sandelin A, Suzuki H, Wei CL, Koseki H, Hasegawa Y, Forrest AR, Carninci P, FANTOM Consortium. 2014. Deep transcriptome profiling of mammalian stem cells supports a regulatory role for retrotransposons in pluripotency maintenance. Nat Genet 46:558–566. doi:10.1038/ng.2965. 24777452

[28] Manghera M, Ferguson-Parry J, Lin R, Douville RN. 2016. NF-kappaB and IRF1 induce endogenous retrovirus K expression via interferon-stimulated response elements in its 5’ long terminal repeat. J Virol 90:9338–9349. doi:10.1128/JVI.01503-16. 27512062 PMC5044829

[29] Knössl M, Löwer R, Löwer J. 1999. Expression of the human endogenous retrovirus HTDV/HERV-K is enhanced by cellular transcription factor YY1. J Virol 73:1254–1261. doi:10.1128/JVI.73.2.1254-1261.1999. 9882329 PMC103948

[30] Katoh I, Mirova A, Kurata S, Murakami Y, Horikawa K, Nakakuki N, Sakai T, Hashimoto K, Maruyama A, Yonaga T, Fukunishi N, Moriishi K, Hirai H. 2011. Activation of the long terminal repeat of human endogenous retrovirus K by melanoma-specific transcription factor MITF-M. Neoplasia 13:1081–1092. doi:10.1593/neo.11794. 22131883 PMC3223611

[31] Wang Y, Chen D, Qian H, Tsai YS, Shao S, Liu Q, Dominguez D, Wang Z. 2014. The splicing factor RBM4 controls apoptosis, proliferation, and migration to suppress tumor progression. Cancer Cell 26:374–389. doi:10.1016/j.ccr.2014.07.010. 25203323 PMC4159621

[32] Foroushani AK, Chim B, Wong M, Rastegar A, Smith PT, Wang S, Barbian K, Martens C, Hafner M, Muljo SA. 2020. Posttranscriptional regulation of human endogenous retroviruses by RNA-binding motif protein 4, RBM4. Proc Natl Acad Sci USA 117:26520–26530. doi:10.1073/pnas.2005237117. 33020268 PMC7585035

[33] Fuentes DR, Swigut T, Wysocka J. 2018. Systematic perturbation of retroviral LTRs reveals widespread long-range effects on human gene regulation. Elife 7:e35989. doi:10.7554/eLife.35989. 30070637 PMC6158008

[34] Babaian A, Mager DL. 2016. Endogenous retroviral promoter exaptation in human cancer. Mob DNA 7:24. doi:10.1186/s13100-016-0080-x. 27980689 PMC5134097

[35] Thompson PJ, Macfarlan TS, Lorincz MC. 2016. Long terminal repeats: from parasitic elements to building blocks of the transcriptional regulatory repertoire. Mol Cell 62:766–776. doi:10.1016/j.molcel.2016.03.029. 27259207 PMC4910160

[36] Grow EJ, Flynn RA, Chavez SL, Bayless NL, Wossidlo M, Wesche DJ, Martin L, Ware CB, Blish CA, Chang HY, Pera RA, Wysocka J. 2015. Intrinsic retroviral reactivation in human preimplantation embryos and pluripotent cells. Nature 522:221–225. doi:10.1038/nature14308. 25896322 PMC4503379

[37] Jeeninga RE, Hoogenkamp M, Armand-Ugon M, de Baar M, Verhoef K, Berkhout B. 2000. Functional differences between the long terminal repeat transcriptional promoters of human immunodeficiency virus type 1 subtypes A through G. J Virol 74:3740–3751. doi:10.1128/jvi.74.8.3740-3751.2000. 10729149 PMC111883

[38] Levine AJ. 1997. p53, the cellular gatekeeper for growth and division. Cell 88:323–331. doi:10.1016/s0092-8674(00)81871-1. 9039259

[39] Wei CL, Wu Q, Vega VB, Chiu KP, Ng P, Zhang T, Shahab A, Yong HC, Fu Y, Weng Z, Liu J, Zhao XD, Chew JL, Lee YL, Kuznetsov VA, Sung WK, Miller LD, Lim B, Liu ET, Yu Q, Ng HH, Ruan Y. 2006. A global map of p53 transcription-factor binding sites in the human genome. Cell 124:207–219. doi:10.1016/j.cell.2005.10.043. 16413492

[40] Stiewe T, Haran TE. 2018. How mutations shape p53 interactions with the genome to promote tumorigenesis and drug resistance. Drug Resist Updat 38:27–43. doi:10.1016/j.drup.2018.05.001. 29857816

[41] Wang T, Zeng J, Lowe CB, Sellers RG, Salama SR, Yang M, Burgess SM, Brachmann RK, Haussler D. 2007. Species-specific endogenous retroviruses shape the transcriptional network of the human tumor suppressor protein p53. Proc Natl Acad Sci USA 104:18613–18618. doi:10.1073/pnas.0703637104. 18003932 PMC2141825

[42] Kaya-Okur HS, Wu SJ, Codomo CA, Pledger ES, Bryson TD, Henikoff JG, Ahmad K, Henikoff S. 2019. CUT&Tag for efficient epigenomic profiling of small samples and single cells. Nat Commun 10:1930. doi:10.1038/s41467-019-09982-5. 31036827 PMC6488672

[43] Tao X, Feng S, Zhao T, Guan X. 2020. Efficient chromatin profiling of H3K4me3 modification in cotton using CUT&Tag. Plant Methods 16:120. doi:10.1186/s13007-020-00664-8. 32884577 PMC7460760

[44] Bouaoun L, Sonkin D, Ardin M, Hollstein M, Byrnes G, Zavadil J, Olivier M. 2016. TP53 variations in human cancers: new lessons from the IARC TP53 database and genomics data. Hum Mutat 37:865–876. doi:10.1002/humu.23035. 27328919

[45] Hollstein M, Sidransky D, Vogelstein B, Harris CC. 1991. p53 mutations in human cancers. Science 253:49–53. doi:10.1126/science.1905840. 1905840

[46] Grossman RL, Heath AP, Ferretti V, Varmus HE, Lowy DR, Kibbe WA, Staudt LM. 2016. Toward a shared vision for cancer genomic data. N Engl J Med 375:1109–1112. doi:10.1056/NEJMp1607591. 27653561 PMC6309165

[47] Hubley R, Finn RD, Clements J, Eddy SR, Jones TA, Bao W, Smit AF, Wheeler TJ. 2016. The Dfam database of repetitive DNA families. Nucleic Acids Res 44:D81–D89. doi:10.1093/nar/gkv1272. 26612867 PMC4702899

[48] Kent WJ. 2002. BLAT—the BLAST-like alignment tool. Genome Res 12:656–664. doi:10.1101/gr.229202. 11932250 PMC187518

[49] Chen C, Chen H, Zhang Y, Thomas HR, Frank MH, He Y, Xia R. 2020. TBtools: an integrative toolkit developed for interactive analyses of big biological data. Mol Plant 13:1194–1202. doi:10.1016/j.molp.2020.06.009. 32585190

[50] Hall TA. 1999. BioEdit: a user-friendly biological sequence alignment program for Windows 95/98/NT. Nucleic Acids Symp Ser 41:95–98.

[51] Kumar S, Stecher G, Li M, Knyaz C, Tamura K. 2018. MEGA X: Molecular Evolutionary Genetics Analysis across computing platforms. Mol Biol Evol 35:1547–1549. doi:10.1093/molbev/msy096. 29722887 PMC5967553

[52] Letunic I, Bork P. 2021. Interactive Tree Of Life (iTOL) v5: an online tool for phylogenetic tree display and annotation. Nucleic Acids Res 49:W293–W296. doi:10.1093/nar/gkab301. 33885785 PMC8265157

[53] McLean CY, Bristor D, Hiller M, Clarke SL, Schaar BT, Lowe CB, Wenger AM, Bejerano G. 2010. GREAT improves functional interpretation of cis-regulatory regions. Nat Biotechnol 28:495–501. doi:10.1038/nbt.1630. 20436461 PMC4840234

[54] Huang DW, Sherman BT, Tan Q, Collins JR, Alvord WG, Roayaei J, Stephens R, Baseler MW, Lane HC, Lempicki RA. 2007. The DAVID Gene Functional Classification Tool: a novel biological module-centric algorithm to functionally analyze large gene lists. Genome Biol 8:R183. doi:10.1186/gb-2007-8-9-r183. 17784955 PMC2375021

[55] Ashburner M, Ball CA, Blake JA, Botstein D, Butler H, Cherry JM, Davis AP, Dolinski K, Dwight SS, Eppig JT, Harris MA, Hill DP, Issel-Tarver L, Kasarskis A, Lewis S, Matese JC, Richardson JE, Ringwald M, Rubin GM, Sherlock G. 2000. Gene ontology: tool for the unification of biology. The Gene Ontology Consortium. Nat Genet 25:25–29. doi:10.1038/75556. 10802651 PMC3037419

[56] Robinson JT, Thorvaldsdóttir H, Winckler W, Guttman M, Lander ES, Getz G, Mesirov JP. 2011. Integrative genomics viewer. Nat Biotechnol 29:24–26. doi:10.1038/nbt.1754. 21221095 PMC3346182

