# Supplementary material for: p53 Binding Sites in Long Terminal Repeat 5Hs (LTR5Hs) of Human Endogenous Retrovirus K Family (HML-2 Subgroup) Play Important Roles in the Regulation of LTR5Hs Transcriptional Activity
Source: Microbiol Spectr. 2022 Jul 18;10(4):e00485-22. doi: 10.1128/spectrum.00485-22 (PMC9430305; doi:10.1128/spectrum.00485-22)
Supplement: Supplemental file 1 — Fig. S1-S3. Download spectrum.00485-22-s0001.pdf, PDF file, 0.8 MB [file spectrum.00485-22-s0001.pdf]

Supplementary Figure

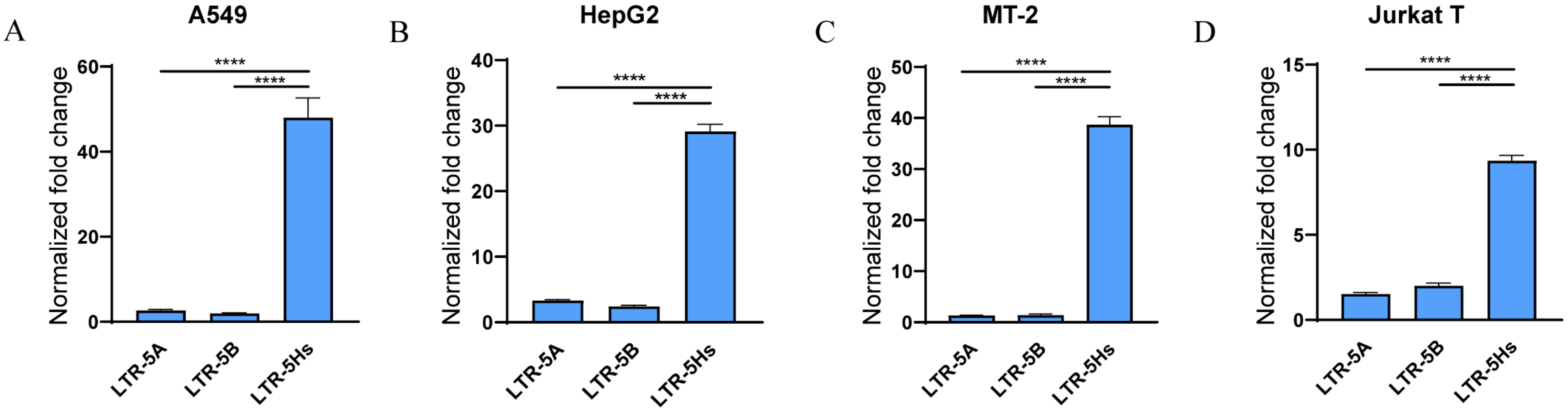

Supplementary Figure 1. Analysis of the promoter activity of LTR5A, LTR5B, and LTR5Hs. LTR5A, LTR5B, LTR5Hs luciferase reporter plasmids and pRenilla-luc-TK were cotransfected into cells. Luciferase activity was measured 48 h after transfection. Then promoter activity of LTR5A, LTR5B, and LTR5Hs in A549 **(A)** and HepG2 **(B)** cells was obtained. LTR5A, LTR5B, and LTR5Hs luciferase reporter plasmids were transfected into cells by electroporation. Luciferase activity was measured 48 h after transfection. Then promoter activity of LTR5A, LTR5B, and LTR5Hs in MT-2 **(C)** and Jurkat T **(D)** cells was obtained. The normalized fold change was obtained as the formula showed in the ***Method*** section. Error bars indicate the standard error for the results of three independent experiments. **** $p < 0.0001$ by one-way ANOVA was considered to indicate statistical significance.

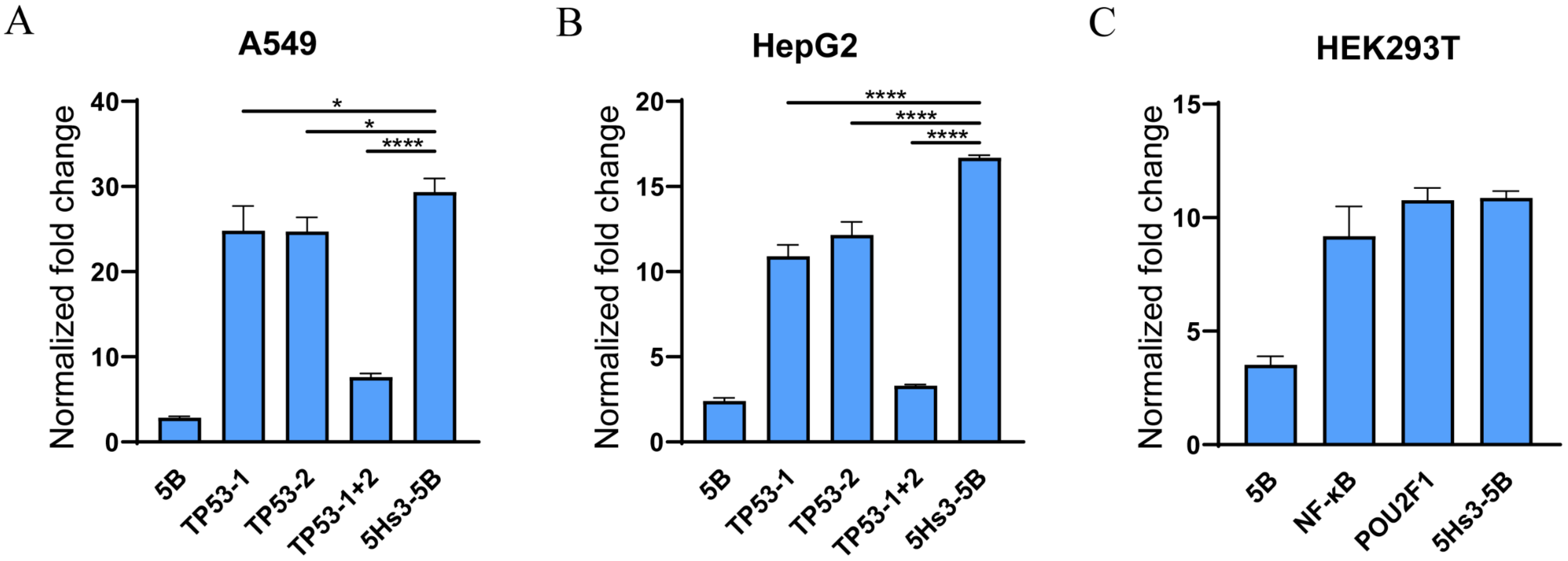

Supplementary Figure 2. *TP53* TFBSs were associated with LTR5Hs activity. Luciferase activity was quantified by using a dual-luciferase reporter assay. Luciferase activity when the sequences were mutated at the *TP53-1* site, the *TP53-2* site, or the *TP53-1* and *TP53-2* sites in A549 **(A)** or HepG2 **(B)** cells**.** 5Hs3-5B luciferase reporter plasmids with mutations in *TP53-1*, *TP53-2*, or *TP53-1* and *TP53-2* binding sites with pRenilla-luc-TK were cotransfected into cells for 24 h. **(C)** Luciferase activity when the *NF-κB* and *POU2F1* sites were mutated. 5Hs3-5B luciferase reporter plasmids with mutations in *NF-κB* or *POU2F1* binding sites, with pRenilla-luc-TK were cotransfected into HEK293T cells for 48 h. The normalized fold change was obtained as the formula showed in the ***Method*** section. Error bars indicate the standard error for the results of three independent experiments. *p < 0.05 and **** p < 0.0001 were considered to indicate statistical significance.

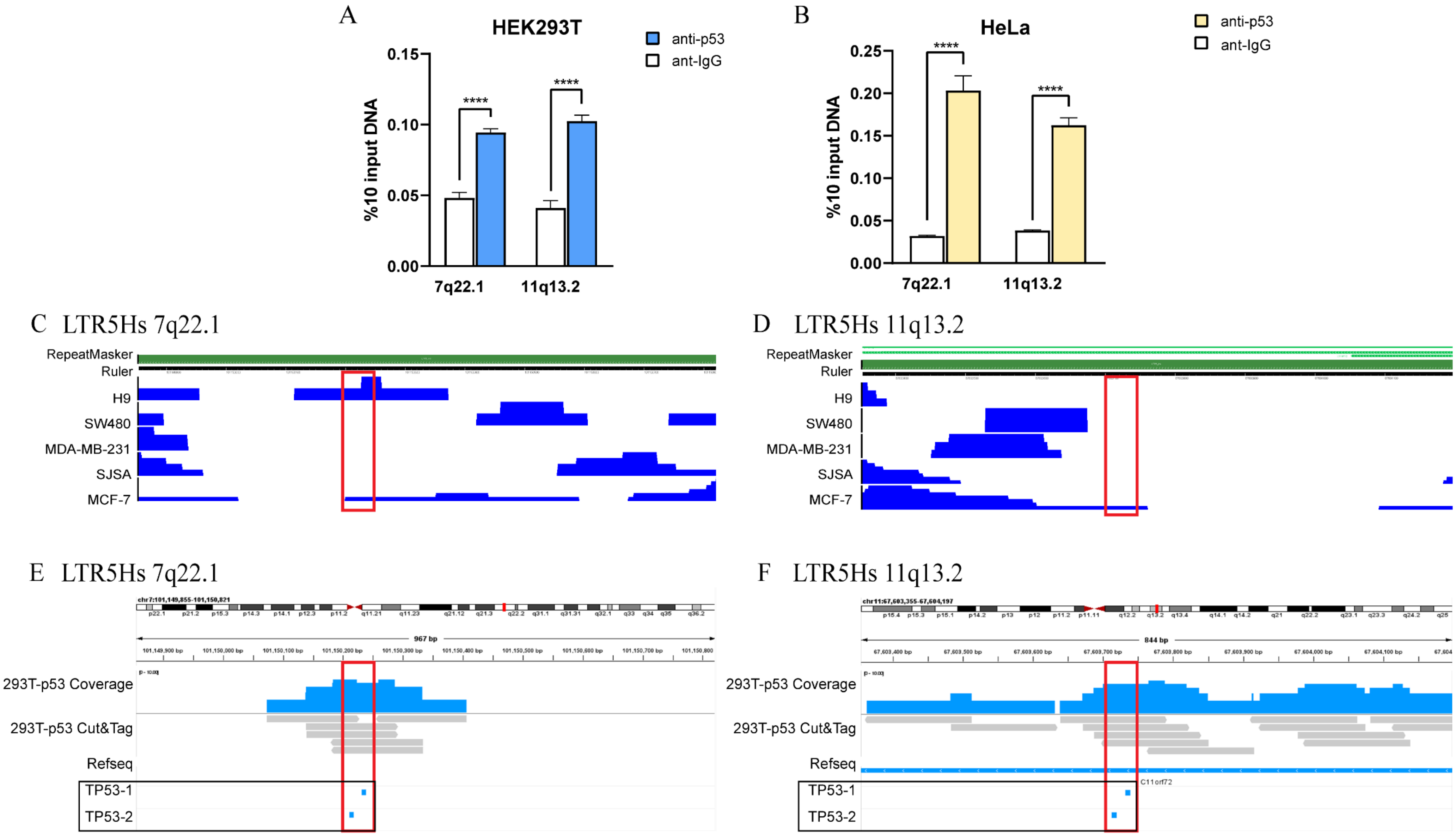

A
HEK293T
anti-p53
ant-IgG
%10 input DNA
0.15
0.10
0.05
0.00
****
****
7q22.1
11q13.2
B
HeLa
anti-p53
ant-IgG
%10 input DNA
0.25
0.20
0.15
0.10
0.05
0.00
****
****
7q22.1
11q13.2
C LTR5Hs 7q22.1
RepeatMasker
Ruler
H9
SW480
MDA-MB-231
SJSA
MCF-7
D LTR5Hs 11q13.2
RepeatMasker
Ruler
H9
SW480
MDA-MB-231
SJSA
MCF-7
E LTR5Hs 7q22.1
chr7:101,149,855-101,150,821
967 bp
293T-p53 Coverage
293T-p53 Cut&Tag
Refseq
TP53-1
TP53-2
F LTR5Hs 11q13.2
chr11:67,603,355-67,604,197
844 bp
293T-p53 Coverage
293T-p53 Cut&Tag
Refseq
C11orf72
TP53-1
TP53-2

Supplementary Figure 3. LTR5Hs elements are transactivated by p53. Chromatin pulled down by antibodies in HEK293T **(A)** and HeLa **(B)** cells was analyzed by qPCR. The error bars indicate the standard error of the mean from three independent transfections. **** $p < 0.0001$ was considered to indicate a significant difference. The p53 protein is enriched in other LTR5Hs elements according to the Cistrome Data Browser database (http://cistrome.org/db/#/). The genomic loci include 7q22.1 **(C)** and 11q13.2 **(D)**. The cell lines included H9 (human embryonic stem cells), SW480 (human colon cancer cells), MDA-MB-231 (human breast cancer cells), SJSA-1 (human osteosarcoma cells), and MCF-7 (human breast cancer cells). CUT&Tag signals across ~ 1000 bp genome regions of LTR5Hs were selected over 7q22.1 **(E)** and 11q13.2 **(F)** were visualized with IGV software. The red box represents the p53 binding region, the blue dot in the black box represents the *TP53-1* and *TP53-2* binding sites.
